# How Many Gammas? Redefining Hippocampal Theta-Gamma Dynamic During Spatial Learning

**DOI:** 10.3389/fnbeh.2022.811278

**Published:** 2022-02-01

**Authors:** Matthieu Aguilera, Vincent Douchamps, Demian Battaglia, Romain Goutagny

**Affiliations:** ^1^Laboratoire de Neurosciences Cognitives et Adaptatives (LNCA), Faculté de Psychologie, Université de Strasbourg, Strasbourg, France; ^2^Institut de Neurosciences des Systèmes, CNRS, Aix-Marseille Université, Marseille, France; ^3^University of Strasbourg Institute for Advanced Study (USIAS), Strasbourg, France

**Keywords:** hippocampus, oscillations, spatial cognition, navigation, complexity, spatial learning

## Abstract

The hippocampal formation is one of the brain systems in which the functional roles of coordinated oscillations in information representation and communication are better studied. Within this circuit, neuronal oscillations are conceived as a mechanism to precisely coordinate upstream and downstream neuronal ensembles, underlying dynamic exchange of information. Within a global reference framework provided by theta (θ) oscillations, different gamma-frequency (γ) carriers would temporally segregate information originating from different sources, thereby allowing networks to disambiguate convergent inputs. Two γ sub-bands were thus defined according to their frequency (slow γ, 30–80 Hz; medium γ, 60–120 Hz) and differential power distribution across CA1 dendritic layers. According to this prevalent model, layer-specific γ oscillations in CA1 would reliably identify the temporal dynamics of afferent inputs and may therefore aid in identifying specific memory processes (encoding for medium γ vs. retrieval for slow γ). However, this influential view, derived from time-averages of either specific γ sub-bands or different projection methods, might not capture the complexity of CA1 θ-γ interactions. Recent studies investigating γ oscillations at the θ cycle timescale have revealed a more dynamic and diverse landscape of θ-γ motifs, with many θ cycles containing multiple γ bouts of various frequencies. To properly capture the hippocampal oscillatory complexity, we have argued in this review that we should consider the entirety of the data and its multidimensional complexity. This will call for a revision of the actual model and will require the use of new tools allowing the description of individual γ bouts in their full complexity.

## Introduction

The ability to represent the surrounding space is crucial for most evolved animals and is at the core of the ability to navigate in the environment, looking out for food, shelter, or other behaviorally relevant locations. For an organism to effectively navigate, it should possess the cognitive representations of critical regions in their environment (e.g., nest locations and food locations), to recall these regions when the need arises, and the means to exploit relations between such regions and their immediate position. In other words, the navigating agent constantly needs to compare current sensory inputs (i.e., encoding of current information) with stored memories (i.e., retrieval of past information). These two seemingly opposed processes (encoding vs. retrieval) are thought to be mediated by two segregated areas of the medial temporal lobe: the hippocampal CA3 region and the entorhinal cortex (EC). Hippocampal CA3, through its massive recurrent network, would support retrieval of past memories (Rolls, 2018), whereas the EC (and more precisely its medial part; MEC) would support encoding of current sensory information (Brun et al., 2002, 2008; Fyhn et al., 2004; Hafting et al., 2005). These two regions in turn project to hippocampal region CA1, which is thought to act as a comparator to determine if ongoing sensory inputs represent new information that needs to be stored (Hasselmo et al., 2002). How does CA1 integrate these different inputs while minimizing interference? Current hypotheses suggest a critical role for brain oscillations in the selective routing of information (Fries, 2009). During spatial navigation, the hippocampus mainly exhibits theta (θ) and gamma (γ) oscillations. It is now accepted that hippocampal γ oscillations can be segregated into slow and medium (or fast, depending on the authors) γ rhythms, each originating from different brain regions and subserving different cognitive functions (Colgin et al., 2009; Schomburg et al., 2014). More recently, studies refining the time scale of analysis have shown that this model might be too simplistic, with a greater variability than initially expected. By putting in perspective these different studies, we have argued in this review that tackling this variability is needed to fully characterize the hippocampal θ-γ dynamic.

## The γ Sub-Bands Model: A Suitable Framework to Understand Hippocampal Computation

Excellent reviews on the cellular mechanisms responsible for hippocampal θ (Buzsáki, 2002) and γ oscillations (Buzśaki and Wang, 2012) have already been published and fell outside the scope of the present review (see also Wang, 2010, for a comprehensive survey of the modeling literature).

Neuronal oscillations are conceived as a mechanism to precisely coordinate upstream and downstream neuronal ensembles, underlying the dynamic exchange of information (Fries, 2009). In the medial temporal lobe, it is proposed that, within a global reference framework provided by θ oscillations, different γ-frequency carriers would temporally segregate information originating from different sources, thereby allowing a target “reader” area to disambiguate convergent inputs (Buzsáki, 2010). As such, hippocampal CA1 γ oscillations, although initially described as forming a single wide frequency band (40–100 Hz; Bragin et al., 1995), were later dissociated into two sub-bands according to their frequency (i.e., slow γ, 25–55 Hz; fast γ, 65–140 Hz), and their phase of appearance related to pyramidal layer θ oscillations (i.e., early phase of the descending part for slow γ and trough for fast γ; Colgin et al., 2009). The fact that bursts of slow γ were associated with increased coherence between CA3 and CA1, whereas fast γ was associated with increased coherence between the MEC and CA1, prompted the authors to suggest that these two independent γ rhythms would selectively “route information” in the hippocampal entorhinal network (Colgin et al., 2009). Building on this framework and on the proposed specific role of CA3 and the MEC in memory processes, the same authors further proposed that these two γ rhythms in CA1 might subserve different cognitive operations, i.e., slow γ would be important for memory retrieval, whereas fast γ would support memory encoding (Colgin and Moser, 2010). While appealing in its simplicity, this model nevertheless carries some caveats. First, while the phase separation of inputs relative to θ oscillation would indeed allow for a separation of the information (Fries, 2009), the reported phase of fast γ does not fit with the “separate phases of encoding and retrieval (SPEAR)” model proposed by Hasselmo et al. (2002). Second, by using single-site recording in the CA1 pyramidal layer, Colgin et al. (2009) were not able to isolate the source of the slow- and fast-γ oscillations. Indeed, one should expect slow γ to be prominent in the CA1 *stratum radiatum* (str.rad), the input of CA3 through the Schaffer collaterals, and fast γ in the CA1 *stratum lacunosum-moleculare* (str.lm), the inputs of the MEC layer 3 through the temporo-ammonic pathway. Finally, while slow and fast γ differentially modulate place cells sequences according to the purported role of each γ rhythm (prospective vs. retrospective coding; Bieri et al., 2014), the authors never actually performed navigation task requiring allocentric memory [open field in Colgin et al. (2009) and linear track in Bieri et al. (2014)].

To fill these gaps, Schomburg et al. (2014) performed high-density multisite recording covering most layers of CA1 to CA3 and dentate gyrus (DG) regions along the transverse axis of the hippocampus in rats navigating in a linear track, a T maze, or an open field. Using a powerful source separation technique and focusing on the hippocampal CA1 area (independent component analysis; Fernández-Ruiz and Herreras, 2013), they were able to identify three γ independent components (ICs). The first component with a strong current sink was localized in the str.rad (termed rad IC), exhibiting slow-γ oscillations (30–80 Hz), phase-locked to the descending phase of CA1 pyramidal θ. The second γ component with a strong current sink was localized to the str.lm (termed lm IC), exhibiting mid-γ oscillations (60–120 Hz), phase-locked to the peak of CA1 pyramidal θ. Finally, the third component with a current source was localized in the CA1 pyramidal layer (termed CA1 pyr IC), exhibiting fast γ (>140 Hz), phase-locked to the through of CA1 pyramidal θ. Based on the location of the current sink/sources and single-unit recordings (in CA1, CA3, and the MEC), the authors proposed that slow γ would represent a communication channel between CA3 and CA1, whereas mid-γ would aid communication between the MEC and CA1. Importantly, there is a clear difference in the θ phase between the mid-γ reported by Schomburg et al. (2014) and the corresponding fast γ reported by Colgin et al. (2009), which can be due in part to the lack of source localization in the study by Colgin et al. Nevertheless, the relative phase of slow and medium γ in the Schomburg et al. (2014) study is coherent with the SPEAR model (Hasselmo et al., 2002). Do these different γ components subserve different cognitive operations? To answer this question, Schomburg and colleagues characterized the dynamics of the γ components during different phases of a T-maze task. They showed that the θ-γ coupling strength of the rad IC selectively increased in the center arm of the maze, a place where memory recall is expected (i.e., in order to guide subsequent behavior). Recently, Fernández-Ruiz et al. (2021) extended this concept to the DG. Using the same decomposition method, they characterized three independent components, namely, a slow-γ IC (30–50 Hz) in the outer molecular layer of the DG, a mid-γ IC (60–80 Hz) in the inner molecular layer of the DG, and a fast-γ IC (100–150 Hz) in the middle molecular layer of the DG, coming from lateral EC associational and/or commissural, and MEC inputs, respectively. During spatial learning, fast-γ oscillations synchronize the MEC and DG, while during object learning, slow-γ oscillations synchronize the LEC and DG. To assess causality, the authors performed γ-frequency optogenetic perturbation of MEC and LEC. This led to reduced DG layer-specific fast- and slow-γ sub-bands and to learning impairments in a spatial and object learning task, respectively.

Altogether, these seminal studies set the stage for what we decided to call the “sub-bands model.” The premise of this model is that if there are different rhythms generated by different brain regions, they must subserve different cognitive operations. However, a problem with influential models is that they tend to inform research in the field, biasing the interpretation of results and narrowing the spectrum of hypotheses that could be considered, e.g., to explain disruptions of function in pathology. For example, a decrease in hippocampal slow-γ power observed in several rodent models of Alzheimer’s disease (Gillespie et al., 2016; Iaccarino et al., 2016; Mably et al., 2017) was linked to retrieval impairment (Mably and Colgin, 2018; Etter et al., 2019) in accordance to the purported role of those oscillations in memory retrieval.

## A Suitable Model, But Surely Too Restrictive

**FIGURE 1:**
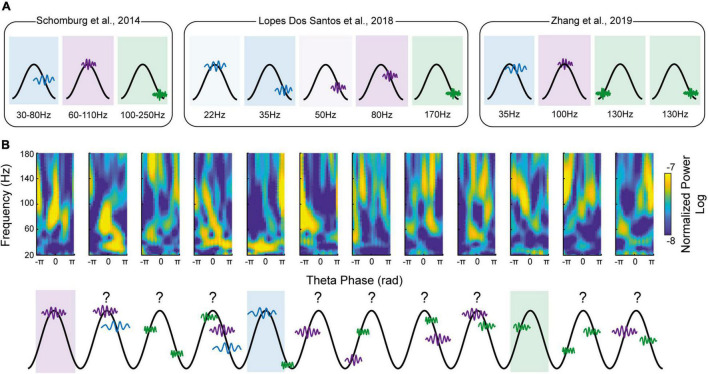
Decomposing theta-gamma oscillations in discrete motifs do not capture the full gamma diversity. **(A)** Schematic representations of the main theta-gamma motifs found in the literature. Gamma frequency is color-coded (blue corresponding to slow-gamma, purple to mid-gamma and green to fast-gamma). Note the inflation of the different motifs since the initial proposal by Colgin et al. (2009) (from 2 in Colgin et al., 2009 to 5 in Lopes-dos-Santos et al., 2018). **(B)** Top: Raw example of theta-gamma motifs found in hippocampal CA1 pyramidal layer (reproduced from Figure 1B of Zhang et al., 2019 https://doi.org/10.7554/eLife.44320.002). Bottom: Schematic representation of the above theta-gamma motifs. Note that only few theta cycles present a prototypic motif as described in **(A)**. In fact, most of the theta cycles can be described as a weighted combination of the different prototypic motifs.

As stated in the “Introduction” section, a navigating agent constantly needs to compare current sensory inputs (i.e., encoding of current information) with stored memories (i.e., retrieval of past information). To gain a better temporal resolution, one can study γ dynamic at the θ time-scale level (Dvorak et al., 2018; Lopes-dos-Santos et al., 2018; Zhang et al., 2019), a proposed unit of computation (Lisman, 2005; Lisman and Buzsáki, 2008). By using various methods of γ detection in a θ cycle by cycle manner (γ detector reported by Dvorak et al., 2018, Ensemble Empirical Mode Decomposition of a γ signal reported by Lopes-dos-Santos et al., 2018 and unsupervised clustering reported by Zhang et al., 2019), these studies showed a more complex landscape than initially proposed (with an increasing number of θ-γ motifs, with up to 5 prototypic motifs reported by Lopes-dos-Santos et al., 2018; Figure 1). Overall, all the studies agreed on the presence of at least three different γ oscillations, similar to the definition put forward by Schomburg et al. (2014) (Figure 1). Collectively, they support the concept that different γ frequencies subserve different cognitive operations by channeling information in specific pathways (refer to Zhou et al., 2019, for a critical view on the existence of a “real” slow-γ oscillation in the hippocampal network). At the first sight, they seem to consolidate the current γ sub-bands model. However, they also all report high diversity of coupling patterns across θ cycles, with most of the cycle containing multiple γ events. In other words, each θ cycle can simultaneously contain slow- and medium-γ events (Figure 1). This diversity was always mentioned, but surprisingly not properly studied, as they all acknowledge restricting analyses to either the highest amplitude γ events (Dvorak et al., 2018) or the one fitting the best canonical clustered results (Lopes-dos-Santos et al., 2018; Zhang et al., 2019). As such, they analyzed only a part of the available landscape of θ-γ motif (36% of all θ cycles reported by Lopes-dos-Santos et al., 2018). What does that imply in terms of local computation? It was indeed assumed that each θ cycle would subserve a specific function based on the associated dominant γ oscillation: in CA1, θ cycles with slow γ would be “retrieval cycles,” whereas cycles with mid-γ would be “encoding cycles” (Colgin et al., 2009; Bieri et al., 2014; Schomburg et al., 2014). The fact that θ cycles mostly contain multiple, low-amplitude, different γ events complexifies this hypothesis (Bagur and Benchenane, 2018). What is the role of a θ cycle with concomitant, same-amplitude slow and medium γ? Can one θ cycle promote different cognitive operations? As an example of possible complexity, Lopes-dos-Santos and colleagues have shown that each θ-nested spectral component (tSC, equivalent of a specific θ-γ motif) represents a distinct spiking dynamic of distinguishable cell ensembles (Lopes-dos-Santos et al., 2018). Since each θ cycle does not present a single tSC but a weighted combination of multiple tSC, what will be the output of such θ cycles (e.g., multiple assemblies co-firing and sequential ordering of different assemblies).

Most, if not all, of the aforementioned studies have focused on the interaction between multiple γ and single θ oscillations (CA1 pyramidal θ). However, it was recently shown that θ oscillations themselves in the dorsal hippocampus are not a unitary process. Using ICA decomposition, López-Madrona et al. (2020) identified three independent θ ICs contributed by different synaptic pathways, namely, the first in the str.rad, the second in the str.lm, and the third in the mid-molecular layer of the DG. Thus, as there are multiple γ, there may also be multiple θ, opening the way to a potential combinatorial explosion of the number of possible θ-γ configurations.

As a concluding, near tautological, remark, we would like to stress that any method for the unsupervised extraction of classes of oscillatory events will end up finding them. The exact number of identified patterns will depend on the specificities of the experimental dataset and algorithm used but will always nevertheless remain a discrete integer number as the applied methods are designed to do so. In front of the inflation of the number of possibly relevant θ-γ patterns exhibited by the recent literature, and the diversity of γ sub-bands definition across aforementioned studies (see Zhou et al., 2019), it may be legitimate to wonder whether the paradigm of looking for discrete classes of events is well-grounded. From a complex dynamics perspective, a neural circuit with recurrent excitatory and inhibitory interconnections is supposed to give rise to oscillations that are not well-behaved and tuned metronomes but can fluctuate in frequency as a function of noisy background inputs (Brunel and Wang, 2003; Brunel and Hansel, 2006). Such stochastic-like oscillations, despite their highly transient and irregular nature, can still be fit for functions as selective information routing, thanks to emergent self-organization mechanisms (Palmigiano et al., 2017). It may thus be that the diversity of θ-γ oscillatory patterns displayed by neural recordings is not the manifestation of the coexistence of multiple, discrete generation mechanisms or sources, but instead the unique, diverse output of a common underlying circuit dynamics, non-linear and complex in nature.

It is noted that a similar debate has also occurred in the literature concerning the diversity of cortical interneurons, that may exist as a large number of discrete types with different functions (Markram et al., 2004; Burkhalter, 2008) and form an interneuron continuum (Parra et al., 1998) or a structured continuum with smooth tendencies (Battaglia et al., 2013).

## Conclusion and Perspectives: Is θ-γ Landscape Random or Complex?

In this review, we have argued that the current model of hippocampal θ-γ oscillations might not capture the complexity of CA1 θ-γ interactions despite the evident appeal of its simplicity and the functional link to memory processes. Indeed, rather than containing a given γ event, most of the θ cycles contain multiple, low-amplitude, γ bouts. Furthermore, many of the observed γ frequencies do not fit well into a classification involving only a few discrete γ types. Should these low-amplitude events be dismissed as noise? To properly describe hippocampal oscillatory complexity, we believe the entirety of the data should be taken into consideration (without assuming that the strongest γ events are the only ones carrying information). This will require the use of new tools that do not assume *a priori* that certain classes of γ rhythms exist but instead enable the description of individual γ bouts in their full complexity. Individual oscillatory events may have wildly fluctuating frequency, amplitude, and phase with respect to ongoing θ. However, these fluctuations could still be correlated to behavior and memory processing but in a collective and synergistic manner. Individual features of oscillatory activity may be only weakly informative about behavior because of their apparent randomness. However, multiple features taken together may still carry relevant information that individual features do not (Wibral et al., 2017). Such a situation may occur if the observed oscillatory events do not arise in all theoretically possible configurations of features but are sampled on a low-dimensional manifold in state space (Chaudhuri and Fiete, 2016). This representation would constrain dynamic trajectories, creating interdependencies between them, possibly modulated by context. In such a view, there would not be discrete classes of oscillatory events but a lower-dimensional space of possible modes of oscillation that the system can smoothly explore along time, possibly under the biasing influence of the exogenous or endogenous input drive. Machine learning approaches could then be used to learn these manifolds without fully determining oscillatory modes and their relations to behavior so as to decode and extract the complex information hidden in the apparent stochasticity of the observed activity time-series.

## Author Contributions

MA, RG, and DB wrote the manuscript. VD provided critical inputs. All authors contributed to the article and approved the submitted version.

## Conflict of Interest

The authors declare that the research was conducted in the absence of any commercial or financial relationships that could be construed as a potential conflict of interest.

## Publisher’s Note

All claims expressed in this article are solely those of the authors and do not necessarily represent those of their affiliated organizations, or those of the publisher, the editors and the reviewers. Any product that may be evaluated in this article, or claim that may be made by its manufacturer, is not guaranteed or endorsed by the publisher.
